# Injuries in professional women’s elite soccer players in Kosovo: epidemiological injury study

**DOI:** 10.1186/s13102-023-00746-9

**Published:** 2023-10-12

**Authors:** Feim Gashi, Tine Kovačič, Ismet Shalaj, Bekim Haxhiu, Arben Boshnjaku

**Affiliations:** 1 Physiotherapy Program, Faculty of Medicine, Alma Mater Europaea -ECM, Maribor, Slovenia; 2 Physiotherapy Department, Faculty of Medical Sciences, Alma Mater Europaea Campus College Rezonanca, Prishtina, Kosovo; 3 Physiotherapy Department, Faculty for Health Science, University of Ljubljana, Ljubljana, Slovenia; 4 Physiotherapy Program, Faculty of Medicine, University “Fehmi Agani” in Gjakova, 50000 Gjakova, Kosovo

**Keywords:** Injury incidence, Female, Trauma, Overuse, Sports

## Abstract

**Background:**

An emphasis has been given lately towards women’s engagement together with their potential in soccer. As this sport develops with athletes becoming more physically fit and skilled, it is unclear what the consequences in terms of injuries are. Having this in mind, this study aimed to investigate the major injuries that occur in women’s soccer players.

**Methods:**

This descriptive epidemiological study invited all 286 women’s soccer players from the 12 participating women clubs in the Kosovo 1st Soccer League (elite football level) during the 2021/2022 season, out of which 142 from 12 clubs participated. Exposure time for 1000 h of playing and training were recorded in addition to the anthropometric data, playing position, and prior injury history during the end of the season, practice, and match. The exact type of injury, severity, and post-injury recovery time, as well as the circumstances surrounding the injuries, were recorded.

**Results:**

In total 84 injuries were registered with an overall injury ratio (IR) being 3.21 (CI: 2.56, 3.98) injuries/1000 exposure hours. During the competitive season, each player sustained 1.4 injuries on average. IRs were significantly higher during competition (n = 50; IR = 1.57; CI: 1.52, 1.62) compared to training (n = 34; IR = 0.26, CI: 0.25, 0.27). Out of a total of 142 women players, 84 (59.2%) injuries occurred, and no record of injuries was made in 58 (40.8%) players. The overall IR was observed to be 3.21 (CI: 1.24, 3.27), with moderate and severe injuries accounting for 38.1% of total injuries (each), followed by mild (16.7%) and minimal (7.1%) injuries.

**Conclusion:**

The women IR in Kosovo women’s soccer players is low while being circa 11% below the international average. Almost 2 out of every 4 injuries were categorized as traumatic, with the IRs being more than 5-fold larger during games than during training. Additionally, these findings emphasize the higher rate of injuries amongst younger athletes, suggesting caution to be taken by the coaches when planning for the match. The collected data may help coaches and trainers create more targeted women’s soccer injury prevention programs.

## Background

A popular sport around the world is soccer, so to perform at the levels required by this sport, one must possess both excellent tactical and physiological levels [1]. Women’s soccer is an extremely demanding sport that requires a high level of strength, power, endurance, agility, and change of direction, all while requiring remarkable tactical skill and precision. Football is one of the sport activities where physical activity, dexterity, and multiple movements are performed with high intensities up to the maximum [2]. Women’s football has recently been growing and day by day the level is increasing, thus reaching new levels with time going by [3]. International Federation of Association Football (FIFA) data on the 2019 Women’s World Cup in France shows an increase in competitiveness compared to the 2015 World Cup, resulting amongst others in injuries as well [4]R. The frequency of injuries in this sport increases due to the great desire for the best performance on the field as well as the contacts that occur during the activity [5]. In the last decade, many numerous types of empirical studies are available, which present the characteristics, types, and incidence of injuries in many countries of the world [5–9]. Another point of concern is the many disagreements regarding the most significant factors in the incidence of injuries in soccer players, including anatomical characteristics of women players (Q angle), biomechanical factors, muscle disharmony between quadriceps and hamstrings hormonal factors, body mass index (BMI), neuromuscular fatigue and its consequences, repetitions, age and especially a reduction in the range of motion (ROM) lack of flexibility in the muscles involved [10–12]. Any physical injury of the players that results in removal from the game or training is defined as an injury, while a repeated injury is considered a new injury according to [13]. Injuries of the lower extremities are very frequent in female players, with roughly 60-80% among all injuries being responsible for keeping them away from exercise and play [14–16]. The major types and forms of injuries reported are easily identifiable and almost identically described by many researchers. The most common injuries in sports, qualified as injuries in game or exercises, are either injuries due to trauma (e.g. contact) or overuse [13, 17–20]. The lower extremities are the areas having the highest rate of injuries in elite-level female players, accompanied with knee injuries, ankle, and hip.4 − 1 Several studies report the incidence of injuries in women’s national leagues in Germany, Spain / Netherlands, Norway, and Sweden [3]. In this context, this study aimed to investigate the injury incidence, their types and forms occurring in women’s soccer players from the Elite Division of an upper middle-income European country. The hypothesis was that the occurrence depends on the exposure time, playing position and age of players, with the lower limbs injuries being the most frequent.

## Methods

### Study population

A total of 286 female soccer players out of 12 participating teams from the Kosovo Women’s Soccer League (The elite division of women’s soccer in Kosovo) were invited to participate in the study. Every active and officially registered player, from all the playing positions (goalkeepers, defenders, midfielders and strikers) being part of any of the competing teams within this league was eligible to participate. For statistical issue 142players from 12 teams agreed to participate and signed informed consent.

### Data collection

Data collection was performed in weekly bases during the competitive season 2021/2022. Exposure time of playing and training were recorded by a member of the team’s medical staff or the coach of the team after baseline characteristics of the players (including anthropometric data, playing position, and prior injury history) during the end of the season, practice, and match. The exact type of injury, severity, and post-injury recovery time, as well as the circumstances surrounding the injuries, were recorded. The self-reported dominant, non-dominant or bilateral sites of players were recorded. Specific injury report forms in accordance with the FIFA Medical Assessment and Research Center Consensus FIFA (F-MARC) [21] were applied for this reason. All conditions that prevented a player from participating completely in practice or games were noted. According to this questionnaire the injuries were classified as minor, mild, moderate, and severe for absences from play of 1–3 days, 4–7 days, 8–28 days, and more than 28 days, respectively [21]. The severity of injuries was reported based on this instrument (F-MARC). Teams received a comprehensive information through the study handbook that included examples to help teams understand how to record data [20]. The study employed translated versions of the F-MARC forms and adhered to their recommendations on definitions and data gathering methods in soccer injury studies [21].

### Data analysis

The typical characteristics of our study population, including all the data collected from the used assessment instruments, are described using descriptive statistics (means and standard deviation for continuous variables and frequencies for categorical variables). The injury incidence rate (IR) per 1000 h of exposure and the related 95% confidence intervals (CIs) was determined using Poisson regressions with generalized estimating equations to assess the risk of injury across matches and training [22]. as an increasingly used approach in epidemiological studies related to sports injuries [23]. The IRs will evaluate the impact of age where it has been separately estimated for players assigned to the younger (under 24), middle [24–29], or older (> 30) age groups. Age was included in the Poisson regression as a continuous covariate variable to compensate for any bias associated to age differences between goalkeepers, defenders, midfielders, and strikers for the comparison of injury IRs in all playing positions. The statistical software program IBM SPSS Statistics for Windows, Version 25.0 (Armonk, NY: IBM Corp) was used to conduct all statistical analyses.

## Results

### Anthropometric data

In this study, a total of 142 women players of the elite league were included, from which anthropometric assessments of the players, such as the players’ age, weight, height, and BMI were 20.39 ± 3.44 years, 58, 49 ± 6.02 kg, 1.65 ± 0.06 m, and 21.2 ± 1.8 kg/m^2^. The basic characteristics of the players were composed of 14 goalkeepers (9.9%), 54 defenders (38%), 40 midfielders (28.2%), and 34 forwards (23.9%). The dominant leg was determined in 74 players (52.1%), the left one in 22 (155%) players, and both legs in 46 (32.4%) cases (Table 1).

**Table 1:** Descriptive analysis

	Mean			GK (N = 14)			DF (N = 54)			MDF (N = 40)			ST (N = 34)		
Age (years, SD)	20.39 ± 3.44			19.93 ± 3.14			20.41 ± 3.51			21.13 ± 3.86			19.68 ± 2.82		
Height (m, SD)	1.65 ± 0.6			1.67 ± 0.05			1.66 ± 0.63			1.64 ± 0.003			1.65 ± 0.071		
Weight (kg, SD)	58.49 ± 6.02			61.86 ± 6.10			58.76 ± 6.05			57.78 ± 5.71			75.53 ± 6.04		
BMI (kg/m^2^)	21.20 ± 1.8			21.97 ± 2.35			21.29 ± 1.67			21.29 ± 1.95			20.98 ± 1.87		
Dominant leg (n, %)	R	L	Bil	R	L	Bil	R	L	Bil	R	L	Bil	R	L	Bil
	74 52.1%	22 15.5%	46 32.4%	7	1	6	28	11	15	18	6	16	21	4	9

BMI, body mass index; GK, goalkeeper; DF, defender; MDF, midfielder; ST, strikers; R, right; L, left; Bil, bilateral; SD, standart deviation

### Match and training exposure

A total of 26,123 h of exposure were logged during the course of the whole season, including 3,748 h of played matches and 22,375 h of training. Players attended 157.45 ± 31.60 training sessions and took part in 26.20 ± 6.37 matches on average. 183.96 ± 31.47 h were the resultant mean exposure time.

### Incidence rate of overall injury

During the 2021–2022 season’s observation period, 84 injuries were reported. The injury rate ratio (IRR) after adjusting for total exposure time was 3.21(CI: 2.56, 3.98) injuries per 1000 exposure hours. On average, each player suffered approximately 1.69 injuries during the competitive season. Injury IRRs were significantly higher (n = 84; IRR = 11.39; CI: 7.14, 17.96; p < 0.001) during competition (n = 50; IRR = 13.34; CI: 9.90, 17.59) compared to training (n = 34; IRR = 1.52, CI: 1.05, 2.12). Out of a total of 142 women players, 84 (59.2%) injuries occurred, and no injuries were recorded in 58 (40.8%) players. While 60 (42.3%) the single body parts most frequently injured by injuries in the lower extremities were the knee, thigh, and ankle, whereas joint injuries in the upper extremities and trunk made for 24 (16.9%) of all registered injuries. In addition, the majority of injuries were categorized as mild (absence from play between 1 and 3 days, n-6; 4.2%), moderate (absence from play between 4 and 7 days, n-14; 9.9%), or severe (absence > 28 days, n-32; 22.5%). Smaller groups were categorized as minimal (absence from play between 1 and 3 days, n-6; 4.2%) and medium (absence from play between 4 and 7 days, n-14; 9.9%).

**Table 2:** Injuries by location and severity

Injuries by location and severity					
Severity category					
Injured body part	Minimal (1 to 3 days)	Mild (4 to 7 days)	Moderate (8 to 28 days)	Severe (> 28 days)	**Total**
Head/Face		1 (7.1%)	1 (3.13%)		2 (2.4%)
Neck / Cervical Spine		1 (7.1%)			1 (1.2%)
Lumbar Spine		1 (7.1%)		1 (3.1%)	2 (2.4%)
Pelvis / Sacrum	1 (16.7%)		1 (3.13%)		2 (2.4%)
Shoulder		1 (7.1%)	2 (6.25%)	3 (9.4%)	**6** (**7.1%)**
Elbow				1 (3.1%)	1 (1.2%)
Lower Arm			1 (3.13%)		1 (1.2%)
Wrist		1 (7.1%)	1 (3.13%)	3 (9.4%)	5 (6.0%)
Hand			1 (3.13%)		1 (1.2%)
Finger / Thumb		1 (7.1%)	1 (3.13%)	1 (3.1%)	3 (3.6%)
Hip		1 (7.1%)			1 (1.2%)
Groin			1 (3.13%)		1 (1.2%)
Musculus adductor			2 (6.25%)	1 (3.1%)	3 (3.6%)
Hamstring		1 (7.1%)	3 (9.38%)	1 (3.1%)	5 (6.0%)
Quadriceps			1 (3.13%)		1 (1.2%)
Thigh			1 (3.13%)		1 (1.2%)
Knee	1 (16.7%)	1 (7.1%)	5 (15.63%)	14 (43.8%)	**21** (**25.0%)**
Lower Leg	1 (16.7%)		1 (3.13%)	1 (3.1%)	3 (3.6%)
Achilles Tendon		2 (14.3%)	2 (6.25%)	1 (3.1%)	5 (6.0%)
Ankle	1 (16.7%)		5 (15.63%)	4 (12.5%)	**10** (**11.9%)**
Foot	2 (33.3%)	2 (14.3%)	2 (6.25%)	1 (3.1%)	7 (8.3%)
Toe		1 (7.1%)	1 (3.13%)		2 (2.4%)
Total	6 (7.1%)	14 (16.7%)	32 (38.1%)	32 (38.1%)	84 (100%)

When analysing the injury occurrence based on the body region (Table 2), it can be observed that the majority of injuries are with in the lower extremities (n = 58, 69.2%), including knee (n = 21, 25.0%), ankle (n = 10, 11.9%) and foot (n = 7, 8.3%) as the three most common sites. It should be highlighted that shoulder injuries were the most common type of injury to the upper extremities (7.1%), whereas the dispersion of injuries within the trunk was rather equal in between lumbar (n = 2, 2.4%), pelvis/sacrum (n = 2, 2.4%) and cervical spine (n = 1, 1.2%).

Another interesting finding that was observed in Table 2 was the fact that moderate and severe injuries made up the bulk of the total number of injuries (n = 64, 76.2%), with an equal dispersion in between these two groups (n = 32, 38.1% each).

Table 3 highlights the distribution of injuries by type and severity. Contusions (n = 15, 17.9%), sprains (n = 12, 14.3%), fractures (n = 8, 9.5%) and dislocations (n = 8, 9.5%) injuries that occurred most frequently, representing more than half of all injuries combined (n = 43, 51.2%). The most frequent injuries requiring minimal recovery time were contusions (n = 4, 66.7%), whereas for a mild period of time (4–7 days) the most frequent ones were lacerations / abrasions (n = 3, 21.4%) and tendonitis (n = 3, 21.4%). The most prominent injuries requiring moderate (8 to 28 days) and severe (> 28 days) recovery time were sprains (n = 7, 21.9%), strains (n = 6, 18.8%) and lacerations (n = 4, 12.5%), as well as fractures, dislocations and ligamentous ruptures with or without instability (n = 5, 15.6% in all cases).

**Table 3:** Injuries by type and severity

Type of injury * Severity category Crosstabulation					
Severity category					
Type of injury	Minimal (1-3days)	Mild (4–7 days)	Moderate (8 to 28 days)	Severe (> 28 days)	Total
Fracture		1 (7.1%)	2 (6.3%)	5 (15.6%)	**8 (9.5%)**
Dislocation	1 (16.7%)		2 (6.3%)	5 (15.6%)	**8 (9.5%)**
Rupture of muscle				1 (3.1%)	1 (1.2%)
Ligamentous rupture with instability				5 (15.6%)	5 (6.0%)
Ligamentous rupture without instability			1 (3.1%)	5 (15.6%)	6 (7.1%)
Lesion of meniscus				1 (3.1%)	1 (1.2%)
Sprain		2 (14.3%)	7 (21.9%)	3 (9.4%)	**12 (14.3%)**
Strain		2 (14.3%)	3 (9.4%)	2 (6.3%)	7 (8.3%)
Contusion	4 (66.7%)	1 (7.1%)	6 (18.8%)	4 (12.5%)	**15 (17.9%)**
Tendonitis / Bursitis		3 (21.4%)	3 (9.4%)		6 (7.1%)
Dental Injury		1 (7.1%)			1 (1.2%)
Deep wound		1 (7.1%)	1 (3.1%)		2 (2.4%)
Laceration / Abrasion	1 (16.7%)	3 (21.4%)	4 (12.5%)		**8 (9.5%)**
Others Diagnosis			3 (9.4%)	1 (3.1%)	4 (4.8%)
Total	6 (7.1%)	14 (16.7%)	32 (38.1%)	32 (38.1%)	84 (100%)

### Traumatic vs. overuse injuries

**Fig. 1:**
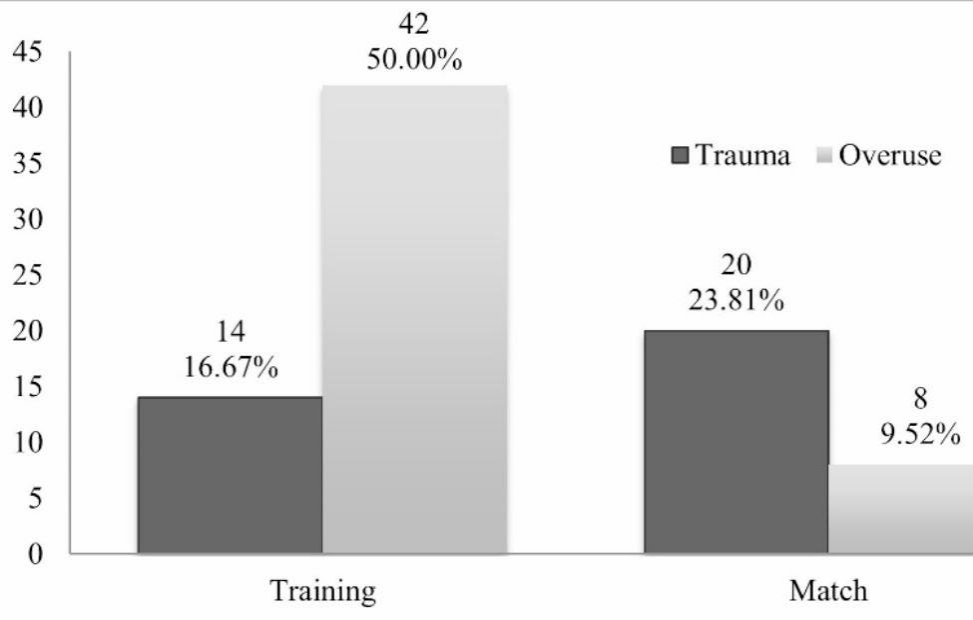
Traumatic and overuse injuries that occur most frequently during training and match

From the total injuries (n = 84; IR = 2.00; CI: 1.24, 3.27), traumatic and overuse ones (n = 56; IR = 2.14; CI: 1.61, 2.78) were the majority (66.7%) and significantly higher (p < 0.05) than the overuse injuries (33.3%; n = 28; IRR = 1.07; CI: 0.71, 1.54), as observed in Fig. 1.

**Fig. 2:**
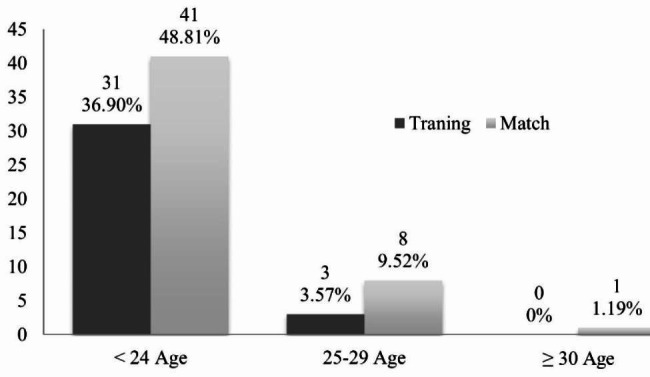
Relative numbers of injuries occurring during training and matches separated by age

Overall number of injuries differed between those occurring in training and those in match (34 versus 50), declining from 36.90% to 48.81% (31 and 41 players respectively) amongst players aged 24-year-old and younger, 3.57% and 9.52% (3 and 8, respectively) in those aged between 25 and 29, and 0% and 1.19% (0 and 1, respectively) in athletes aged 30 years and older (Fig. 2).

**Fig. 3:**
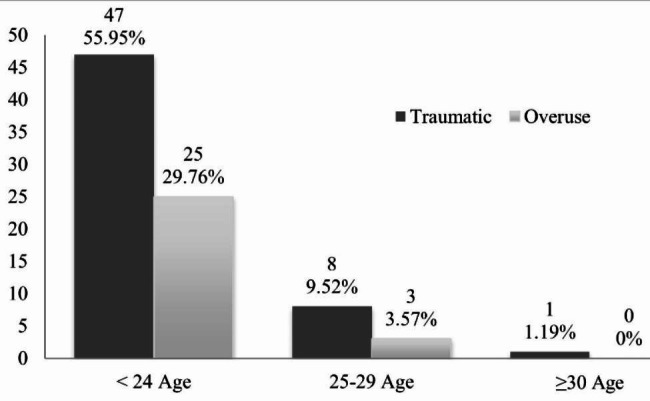
Relative numbers of traumatic and overuse injuries separated by age

Additionally, the number of injuries (both from overuse and traumatic origins) declined from 29.76% to 55.95% (25 overuse and 47 traumatic injuries, respectively) in the age group of 24 years old and below, to 3.57% and 9.52% (3 and 8, respectively) in the 25–29-year-old group, and no injuries and 1.19% (0 and 1, respectively) in the above age group of 30 years and above (Fig. 3).

### Injuries depending on the position playing

A total of 84 injuries were recorded. Amongst those, goalkeepers (n = 9, 10.7%, IR = 1.07; CI = 0.49, 2.03), strikers (n = 17, 20.2%; IR = 2.02; CI = 1.18, 3.24), midfielders (n = 23, 27.4%; IR = 2.74; CI = 1.74, 4.11) represented the most frequently affected groups, followed by defenders (n = 35, 4.17%; IR = 2.47; CI = 1.72, 3.43 of all injuries). Defenders, on average, were the group’s youngest members 20.4 ± 3.52 years, while midfielders were the oldest 20.9 ± 3.56 years. Forwards 20.24 ± 2.70 years old, goalkeepers 20 ± 3.16 years old.

Injury IRRs were significantly higher (IRR = 25.71, CI = 10.87, 54.57, p < 0.001) in goalkeepers (n = 9, 10%0.7) comparing to defenders (n = 35, 41.7%). Yet this was not the case in between strikers (n = 17, 20.2%) and midfielders (n = 23, 27.4%), where no observable differences were significant (IRR = 1.35, CI = 0.69, 2.69 (p = 0.349).

### Injuries depending on age

Players were divided into groups according to their ages: young (< 24 years n = 72, 85.7% IR = 8,57; CI = 6,70, 10.79), middle (24–29 years; n = 11, 13.1% IR = 1,31; CI = 0,65, 2.34) or old (> 29 years older; n = 1, 1.2%). The major findings observed in this context were the significantly higher number of injuries occurring amongst younger women soccer players (IRR = 6.54, CI = 3.43, 13.69 (p < 0.001) in comparison to the “middle” age group (Table 4).

**Table 4:** Injuries depending on age

Age & Injuries				
		Total Injuries (n %)		Total
Age	< 24	72	85.70%	72
	24–29	11	13.10%	11
	≥ 30	1	1.20%	1
Total		84	100%	84

## Discussion

The current study is Kosovo is the first epidemiological study that examined the soccer-related injuries in women soccer elite players. A total of 84 injuries were noticed while monitoring 26,123 h of exposure among 12 clubs and 142 players from the Elite Women’s Soccer League. According to these results, there are 3.21 (CI: 1.24, 3.27) overall IR injuries for every 1000 h of exposure.

### Incidence rate of overall injury

Many studies have been investigating soccer-related injuries played in various major soccer leagues [14, 24–27], many of which show significant differences across countries in the frequency of injuries. In this study, the elite women’s soccer league’s overall injury rate was observed in Kosovo is 3.21 (CI: 1.24, 3.27), which was surprisingly lower than in other studies. In this context, a systematic review and meta-analysis conducted by [24], reported an overall injury incidence in soccer football players being 6.1 injuries /1000 h exposure, out of which a number of match injury incidence (19.2 injuries / 1000 h of exposure) about 6 times higher than training injury incidence rate (3.5 injuries / 1000 h of exposure). However, Horan D and colleagues were in the same line with their findings from the Irish Women’s National League [26], reporting a 7.9 injuries / 1000 h exposure overall injury incidence ratio with an even higher difference (7.5 times) in between match (192 / 1000 h) and training (2.5 / 1000 h). Another recent study from the first division of Spanish Women’s Soccer League [25], reported an injury incidence ration of 3.65 injuries / 1000 h with an even higher match versus training injury incidence ratio (19.02 / 1000 h and 1.70 / 1000 h, respectively). An earlier 2005 study from the German’s National League [14], reported an injury rate of 6.8 injuries / 1000 h of exposure, while being as high as 23.3 / 1000 in match hours and 2.8 / 1000 h of training. Another study from the same year though coming from Swedish female elite soccer players [27] reported an injury ratio 4.6/1000 h of soccer being 13.9 / 1000 h exposure during match and 2.7 / 1000 exposure during training. The differences in between all the findings (including ours within this pool) could be due to the methodological differences, different time periods when the studies were performed etc. However, the very low injury incidence ratio observed within our study might just correspond to the generally lower level of our elite soccer league and on its late internationalization.

The quantity of exposure and the total number of games played in a season have both been linked to injury occurrence because they have an impact on how long players need to regenerate between games [5]. In our study, participating players played an average of 26.20 ± 6.37 matches. Although the best clubs may use more elite players and switch up their lineups more frequently (the differences in average match hours were just 13% and 17%, respectively), the much-reduced match exposure may account for Kosovo’s low injury rate overall. The fact that Kosovo joined FIFA and UEFA extremely late and missing in international competitions, can be linked to the causes for the lesser number of matches played during the season.

Kosovo’s unusually low injury rate may also be explained by the “northern bias” mentioned by [28], According to these articles, teams from northern European regions—which have also undergone more in-depth study—had a higher frequency of injuries than teams from Mediterranean climatic zones. This can be because the climate has an impact on injuries incidence. Higher wind speeds and colder outside temperatures, according to a study on Scottish rugby players, increased the chance of injury [29].

The style of playing may also be attributed to the geographical variations in injury occurrence. It is probable that a more technical and physically less demanding style of play would be related with a lower overall risk of injury, according to [30].

### Types of injuries

In our studies, contusions, sprains, and fractures were the three most common types of injuries, accounting for more than half of all injuries. The most common differential diagnoses included contusions (n = 15–17.9%), sprains (n = 12, 14.3%), fractures, dislocations, and lacerations/abrasions (n = 24, 28.5%). Based on these data, “a re-evaluation of injury prevention program in women’s soccer,” should be performed with the goal of more effectively reducing the most common types of injuries. The statistical analysis and evaluation of the study data revealed a “need for targeted preventive women’s soccer programs especially for sprains, contusions, fractures, dislocations, abrasions to reduce the need for surgical intervention, as well as further research into potential reasons for these observed ratings regarding types and severity as well as location and severity. These findings do not differ from findings of other epidemiological research, such as the one by [31], in which sprains were the most frequent type of game injury and contusions were reported in 21 investigations.

### Injuries in matches vs. training

According to our data, trauma injuries damage that was about two times higher than overuse syndrome (n-28, 33.3% vs. n-56, 66.7%) or exercises (IR = 3.2). Although this result is consistent with earlier research [6]. The dramatically greater rates of injuries in games may be explained by a study by Rahnama and colleagues that investigate at playing behaviours linked to an increased risk of injury in soccer matches [32]. It might be assumed that during matches, these activities happen more frequently and are carried out more forcefully. Additionally, it has been discovered that injuries tend to happen more frequently at the conclusion of each half [5], providing evidence that suggests weariness may play a part in making injuries more likely to happen during games.

### Injuries depending on age and playing position

Soccer injury rates have been the subject of several studies looking at the connection between playing position and injury rates, with varying degrees of success. The age of the players is one element that can contribute to this variability of results. Despite some conflicting data [10], various research has predicted that the frequency of soccer injuries will rise with advancing age [33]. In our study, the injury ratio decreased with age starting with younger players (n = 72, 85.7%), middle players (n = 11, 13.1%), and elderly players (n = 1, 1.2%). According to these findings, injuries are six times more common in the youngest age group, with statistically significant disparities between the young and middle age groups. According to these findings, injuries are six times more common in the youngest age group, with statistically significant disparities between the young and middle age groups. These findings might point to a more aggressive, les of experience and dangerous playing style among young players [20]. To account for any age-related biases when comparing IR injury amongst players who were involved in different playing positions, age was included as a covariate in the Poisson regression analyses due to the considerable differences between age groups. This finding may be connected to the idea that the incidence of injury is highest during games (where most injuries occur) in the regions of the field where possession is most fiercely contested, such as in the defensive areas close to the goal [32]. It’s crucial to remember that the differences weren’t statistically significant and were somewhat slight. Our findings confirm earlier research suggesting that playing position has no bearing on the frequency of injuries [34].

### Limitations

It should be acknowledged as a potential drawback of our investigation that the incidence of injury recorded in this study solely depended on the assessment of members of the medical staff teams. A thorough multidisciplinary medical team might be able to address this gap as different diagnoses could not be independently verified by a single supervising expert. The age distribution of the players was also skewed, with the majority of athletes being 24 years or younger (72/142) and the minority being 29 years or older (11/142).

## Conclusions

This study shows that Kosovo has fewer soccer injuries overall than other European leagues, on average. These outcome can be attributed to the Kosovar players playing in significantly fewer matches than their international counterparts. Nearly two out of every four injuries in soccer were categorized as traumatic, which is consistent with the findings of another epidemiological research. Notwithstanding the fact that some of the findings in women’s soccer players in Kosovo were not in line with other research—particularly related to overall injuries, the larger scale and more extensive detail offered in the current study should give coaches and trainers better insight into the future prevention needs of their women’s soccer players. As strikers, defenders, midfielders, and goalkeepers all have different physical and physiological demands it is of great importance to understand and train for these differences in order to properly prepare women for their specific position and simultaneously reducing the risk of sport injury. The two differential diagnoses that were used most frequently were contusion and sprain, while there were no differences between players playing in different positions, with younger players having much higher injury IRs than middle-aged and older players. This research provides novel data on the frequency, nature, and severity of injuries in a developing European soccer league as it is the first epidemiological assessment of soccer injuries in Kosovo. Future research will use these findings to identify risk factors for the most prevalent differential diagnosis and will work to create specialized preventative strategies.

## Data Availability

The corresponding author can provide the data described in this study upon request.

## Abbreviations

IR: Injury rate
FIFA: International federation of association football
BMI: Body mass index
ROM: Range of motion
F-MARC: FIFA medical assessment and research center consensus
CI: Confidence interval
GK: Goalkeeper
DF: Defender
MDF: Midfielder
ST: Strikers
R: Right
L: Left
Bill: Bilateral
IRR: Injury rate ratio
